# Social relations and health in older people in Spain using SHARE survey data

**DOI:** 10.1186/s12877-022-02975-y

**Published:** 2022-04-04

**Authors:** Mariela Silberman-Beltramella, Alba Ayala, Carmen Rodríguez-Blázquez, Maria João Forjaz

**Affiliations:** 1 Hospital Nuestra Señora de Candelaria, Santa Cruz de Tenerife, Spain; 2 University Institute on Gender Studies, University Carlos III of Madrid, Madrid, Spain; 3 National Center of Epidemiology, Carlos III Institute of Health, Madrid, Spain

**Keywords:** Social relationships, Health, Healthy ageing, Loneliness

## Abstract

**Introduction:**

Social relationships (SR) are an important aspect in the healthy ageing process. The study aimed to describe SR in over-50s in Spain and analyse their association with physical/emotional, functional and cognitive/sensory health variables.

**Methods:**

The study sample was formed by 5583 people representing the Spanish population aged 50 and over, who participated in the sixth wave of the Survey of Health, Ageing and Retirement in Europe (*SHARE*). The variables were divided into socio-demographic aspects and economic, health and SR aspects represented by the number of friends, family members and satisfaction with social network and the Revised UCLA loneliness scale. The health variables were grouped using a main component analysis. Multiple linear regressions were performed between the health components with socio-demographic and SR variables.

**Results:**

67.26% of respondents said they did not feel lonely. The feeling of loneliness was the variable most closely related to the physical and emotional, functional and cognitive and sensory health components. The main SR variable associated to health components was the Revised UCLA Loneliness Scale (standardised beta, *p* < 0.001; *p* < 0.001; and *p* < 0.001, respectively). The number of family members in social network SR variable was also associated with the physical/emotional health (β = 0.09, *p* < 0.001) and cognitive/sensory ability (β = 0.10, *p* = 0.001) components.

**Conclusions:**

The main SR aspect that impacts health status was loneliness. The results of this study suggest the importance of developing public health policies oriented to promoting action on the SR characteristics that enhance older people’s health.

**Supplementary Information:**

The online version contains supplementary material available at 10.1186/s12877-022-02975-y.

## Introduction

In Spain, the proportion of people aged 65 or more was already around one fifth of the population in 2019 [1] and the relative increase in the very old group (i.e., people aged 85 years or more) is projected to be among the highest in Europe. Demographic projections suggest that in 2050, Spain will surpass 36% of people aged 65 years or more. This longevity shown by statistics is redefining the roles and image of older people in many countries, and could be considered as a great challenge for social and health systems, in terms of increase of chronic diseases, dependence and psychosocial consequences, such as loneliness and mental health problems. Loneliness and lack of social support have been found to be high in Spanish older people [2, 3]. For example, Tan et al. showed that 36,7% of Spanish older people reported loneliness, and Cuesta-Lozano et al. found that 83.33% of adults aged 65 years or older reported some degree of loneliness [2, 4]. Some cultural aspects (the importance of family and friends and having a close contact with them), have been suggested as an explanation for these figures.

In this context, the main challenge we face is to understand that this possibility of a longer, more satisfactory life is an achievement for everyone and involves a great opportunity for development in aspects that have not yet been explored by human beings [5]. It is within this framework that social relations (SR) become particularly important, as they are part of those aspects that help people to live life to the full and help to maintain both older people’s physical and emotional health, as well as their functional and cognitive ability.

SR are defined as relations between two or more people when both influence the thoughts, feelings and/or behaviours of the other/s [6] although there is no consensus on their definition. This bond based on communication and mutual respect makes people feel loved and cared for, generating this powerful and protective impact on health. The nature of this bond would explain why people with more satisfactory SR (partner, family, friends, etc.) are in a better state of physical and mental health [7].

National [8, 9] and international research [10–12] has found that the health of older people is closely linked to their SR. House et al. in the 1980s already indicated that “social relationships, or the relative lack thereof, constitute a major risk factor for health rivaling the effects of well-established health risk factors such as cigarette smoking, blood pressure, blood lipids, obesity, and physical activity” [13]. Both Otero-Puime et al. and Holt-Lunstad et al. include two fundamental aspects within SR the social network as a structure (partner, family, friends, etc.); and social support as one of the functions performed by that social network. In particular, some authors [14–16] include the degree of satisfaction as a way of assessing social networks. Valtorta et al. argue that the intensity of SR could be measured by a greater or lesser perception of loneliness [6]. Several authors have measured size based on the number of members (family, friends, etc.) in their social networks.

There are several hypotheses on how SR can influence health. Among them, Cohen and Syme [17] propose two models. The first, the direct effect model, assumes that SR provide direct emotional and behavioural support that was not necessarily intended to be supportive, but may encourage changes towards a healthier lifestyle. The second, buffer effect model [18] suggests the presence of an active social support system that would protect against the harmful effects of stressful events. On the other hand, the model proposed by Berkman et al. [12] speaks of psychosocial mechanisms that would act on physiological, psychological or behavioural pathways that would ultimately produce the health effects.

Research over the last decade has found that SR have influenced health by shortening hospitalisation times [19], improving compliance with medical regimens [20] or increasing the probability of survival by 50% [21]. These social interactions and the size of the social network have been linked to overall health status [22]. In addition, a greater number of friends and number of contacts were associated with less stress and better health [23] A good family and social network can predict the maintenance of functional ability and protect cognitive function [24]. Evidence also points to loneliness as an important risk factor in developing various diseases [25]. This line of thought also applies to SR’s effect on healthy ageing, defined by the WHO as “the process of developing and maintaining functional ability that enables well-being in old age”. This model, where ageing begins the day we are born, encourages us to seek the opportunity to develop our full potential in order to live life to the full. It is here where the SR we establish play a key role in this individual, yet not solitary construction of well-being and fulfilment that we cultivate throughout our lives.

Some authors consider that SR influence an individual’s mental and physical health through mechanisms such as social influence, role-based purpose and meaning, self-esteem, belonging and companionship [26]. This coincides with the perspective of Arias and Iglesias-Parro [27], for whom active ageing is not construed as an end state, but as a process that enables people to achieve goals or desired states (absence of feelings of loneliness, less disability, etc.).

Healthy ageing framework has redefined the life course, bringing a new scenario that is reinventing how people live and how old age is regarded. No longer is it merely a time of retirement and increasing disabilities, but a privileged time where health care, coupled with stronger SR, are the cornerstone that enables people to enjoy these additional years of life in just as productive, versatile and comforting a way as all the previous years.

This study tries to answer the question: what will be the structural and/or functional aspects of social relationships that will be differentially linked to health and active aging? To do this, we intend to combine instruments that measure structural components of the social network (marital status, number of members of the network, family, friends, etc.) with functional aspects such as the perception of the feeling of loneliness, in addition to satisfaction with social network. With the inclusion of adults over 50 years of age, the aim is to broaden the view of time and circumstances (eg, transition to retirement) that accompany active aging, conceiving it as a process that encompasses the entire life cycle.

Therefore, the purpose of this study was to describe the SR of a sample of over-50s in Spain and to analyse their association with physical and emotional, functional and cognitive and sensory health variables, which in turn are important elements of healthy ageing.

## Methods

### Study design and sample

Cross-sectional study conducted in Spain, on a sample from wave 6 collected in 2015, of the *Survey of Health, Ageing and Retirement in Europe (SHARE),* a longitudinal, multidisciplinary study on the health, economic status and social and family networks of more than 140,000 individuals from 27 countries in Europe and Israel. Using multistage sampling, 5583 individuals were selected from a representative sample of the Spanish population aged 50 and over, who were contacted for an interview with both the selected individuals and their partners if they lived in the same household.

Data was collected through Computer-Assisted Personal Interviewing (CAPI).

### Ethics and consent to participate

The present study was conducted according to the guidelines of the Declaration of Helsinki, and approved by the Ethics Committee of Carlos III Institute of Health (reference: CEI PI 62–2019). The SHARE project was reviewed and approved by the Ethics Council of the Max Planck Society. All participants gave their informed consent to be included in the study.

### Measurements

The variables selected are divided into three main groups, socio-demographic variables and level of wealth, health variables and SR variables [28–30].

The following socio-demographic variables were collected: gender, age, marital status (with partner, without partner), years of education, employment status (retired, employed, housewife and others) and wealth, which was calculated by adding several variables (value of primary residence, value of any other real estate, value of bank accounts, bonds, stocks and mutual funds, value of sharing part of a business and value of cars), and then subtracting the mortgage of the primary residence and financial obligations [31, 32].

The physical and emotional health component variables included the number of medicines consumed at least once a week; the number of chronic pathologies calculated from a list of 30 common chronic diseases such as heart attacks, diabetes, stroke, cancer, cataracts, among others. For the self-perceived health variable, *SHARE* uses the US version of the scale based on the SF-36 questionnaire [33]. The EURO-D depression scale [34] consists of 12 items with dichotomous response (yes/no). The total score is made by adding up the positive scores. It is considered as a case of depression if a score of 4 or more is obtained, and 12 is the maximum score indicative of severe depression.

Basic activities of daily living (BADL) [29] data was collected as variables of the functional ability component, where *SHARE* uses the modified version of the scale [35, 36], which includes 6 activities with dichotomous responses (yes/no) for each of the functions. The score ranges from 0 to 6 with only affirmative scores being considered. The higher the score, the harder the person finds it to perform these activities and the lower the mobility. For instrumental activities of daily living (IADL) *SHARE* uses the modified version of the scale [37] which includes 7 activities with dichotomous response (yes/no). Only affirmative answers are added up to calculate the total score, ranging from 0 to 7 points. The lower the score, the less difficulty the person has in performing these activities and the greater the mobility.

Cognitive and sensory ability component variables included sensory problems of hearing, vision and memory [38], rated on a 5-point Likert scale (excellent, very good, good, fair, fair, poor).

The main SR variable was measured with the R-UCLA Short Loneliness Scale [39, 40]. This scale indirectly measures loneliness, consisting of three questions referring to feelings of companionship, exclusion and isolation, answered on a three-point Likert-type scale (often, sometimes, and hardly ever or never). The total score ranges from 3 (not at all lonely) to 9 points (very lonely). There are no cut-off points for the degree of loneliness. The remaining SR variables were the number of family and friends in the network, for which the respondent was asked to provide a list of up to seven people, referred to as “contacts in the respondent’s social network”; and the variable degree of satisfaction the respondent has with the people in his or her social network. It is measured on a single global scale ranging from 0 (totally dissatisfied) to 10 (completely satisfied).

### Data analysis

The data was analysed descriptively (frequency, central tendency and dispersion statistics), using individual weights for ensuring the representativeness of the sample. Following the WHO active aging framework, two researchers reviewed all the variables in SHARE database and assigned them to each of the Active Aging pillars. Then, to reduce the number of health variables, they were grouped using principal component analysis (PCA). All variables were standardised to have the same range of scores. PCA was performed using the principal components method and Varimax rotation. The variable scores were calculated by linear combination of the original variables and the final scores were transformed into a scale of 0 to 100, where a higher component score indicates better health.

Subsequently, differences in component scores in the pooled sample were analysed according to socio-demographic and social relations variables. Student’s t-test or analysis of variance (ANOVA) was used to analyse whether there were differences in means between the different groups of variables.

Finally, multivariate linear regression models were performed where the dependent variables were the calculated health component scores. The independent or explanatory variables are the SR satisfaction variables, the short R-UCLA loneliness scale and the number of friends and relatives in the social networks collected in wave 6. All models were adjusted for wealth and socio-demographic variables (age, sex, marital status, level of education and employment status). In all cases we set an alpha level of comparison of 5% for statistical significance, and a 95% confidence interval (CI). Statistical analyses were performed with Stata 15.

All methods were performed in accordance with the relevant guidelines and regulations.

## Results

The average age of the respondents was 70.28 years old (standard deviation, SD = 10.55) and 53.83% of them were women (Supplementary Table 1). The 50 to 64 year old age group represented 47.41% of the study population. The large majority of the participants had a partner (75.32%) and nearly half of the interviewed population was retired (49.33%).

The degree of satisfaction with the social relationships was considered high by 68.59%. The majority of the respondents (67.26%) said that they did not feel any loneliness. Almost all (95.03%) respondents said that a member of their family was present in social network. However, 77.92% of the participants said that they did not have a friend (Supplementary Table 1).

The PCA identified three components (explained variance: 68.37%). In the first component (physical and emotional health), the following variables were grouped from highest to lowest weight: number of medicines (factor weight: 0.57), number of chronic pathologies (0.56), EURO-D scale (0.39), and perceived health (0.43). In the second component (functional ability) the BADL variables (factorial weight: 0.72), IADL (0.68) were grouped together. Finally, in the third component (cognitive and sensory ability) the following variables were grouped: auditory sensory problems (factor weight: 0.62), vision (0.65), and memory problems (0.40) (Table 1).

**Table 1:** Principal component analysis. Distribution of loads for each of the variables that make up the three health components

	Physical and emotional Health	Functional ability	Cognitive and sensory ability
Limitation of basic activities of daily living	−0.019	0.729	−0.027
Limitation of instrumental activities of daily living	0.018	0.682	0.03
Number of chronic pathologies	0.56	−0.016	− 0.045
EURO-D depression scale (0–12 items)	0.396	0.029	0.033
Self-rated health	0.43	0.013	0.131
Number of medicines	0.576	−0.009	−0.074
Memory problems	0.084	−0.002	0.407
Sensory problems (hearing)	0.007	−0.031	0.62
Sensory problems (sight)	−0.063	0.024	0.65

Note. Explained variance: 68.37%

Table 2 presents the mean scores for each health component according to socio-demographic and SR variables. The physical and emotional health component score was significantly higher in people who did not feel loneliness (74.87) than in those who felt some degree of loneliness (63.17, *p* < 0.001) and in people who had a high degree of satisfaction with social networks (71.08) than in those with a low or moderate degree of satisfaction (48.20, *p* < 0.001). In the functional ability component, significant differences were observed in the perception of loneliness, with a higher score in people who did not feel loneliness (96.03) compared to those who felt some degree of loneliness (91.47, *p* < 0.001) and in the number of friends in social networks, with a higher score in those who had between 1 and 7 friends (95.56) compared to those who had none (93.91, *p* < 0.001). In the cognitive and sensory ability component, people who did not feel loneliness scored higher (54.19) than those who felt some degree of loneliness (46.97, *p* < 0.001).

**Table 2:** Score of each health component in the sample grouped by sociodemographic and SR variables

	Physical and emotional health			Functional ability			Cognitive and sensory ability		
	Mean^b^	SD^c^	*p*-value	Mean^b^	SD^c^	*p*-value	Mean^b^	SD^c^	*p*-value
Gender			< 0.001			< 0.001			0.419
Women	67.36	18.47		90.39	19.68		50.19	17.75	
Men	72.89	15.11		93.74	14.92		51.04	15.83	
Marital Status			< 0.001			< 0.001			0.126
Without a partner	66.23	18.06		87.65	23.27		49.32	18.34	
With a partner	71.59	16.54		93.94	13.96		51.18	16.14	
Job situation			< 0.001			< 0.001			< 0.001
Pensioner	66.25	15.96		90.78	18.76		47.38	15.85	
Employed	81.65	11.62		97.81	1.56		58.61	15.60	
Housewife	65.71	17.81		91.95	16.37		48.34	15.72	
Others	67.04	18.00		87.66	23.46		49.96	17.83	
Age			< 0.001			< 0.001			< 0.001
50 to 64 years	76.96	14.56		96.92	5.98		56.68	15.55	
65 to 79 years	66.85	16.31		96.92	14.48		48.85	15.55	
80 years +	57.01	16.26		75.96	30.55		37.74	16.59	
Satisfaction with social network			< 0.001			0.012			0.07
Low-moderate degree	48.20	20.33		88.12	9.91		31.04	9.99	
High degree	71.08	16.66		94.54	11.85		51.75	16.11	
Revised UCLA Loneliness Scale.			< 0.001			< 0.001			< 0.001
Does not feel loneliness	74.87	15.38		96.03	8.98		54.19	15.69	
Feels some degree of loneliness	63.17	16.51		91.47	15.68		46.97	16.27	
Number of friends in social network			0.088			< 0.001			0.277
No friends	70.37	16.97		93.91	13.19		51.32	16.53	
1 to 7 friends	72.31	15.71		95.56	9.63		52.70	14.91	
Number of family members in social network			0.863			0.644			0.242
No family members	71.07	14.91		94.69	11.91		49.36	14.66	
1 to 5 or more	70.78	16.81		94.25	12.54		51.75	16.26	

Mean and Standard Deviation (SD) = ^b-c^ weighted values

Table 3 shows the multiple linear regression model of the physical and emotional health component. The main SR variable associated with the component was the short R-UCLA loneliness scale (standardised β coefficient = −0.26) followed by the number of relatives in social networks (β = 0.09*),* both variables, *p* < 0.001. This model explained 33% of the variance.

**Table 3:** Multivariate linear regression model for the Physical and Emotional Health, Functional Ability and Cognitive and Sensory ability components

	Physical and emotional health		Functional ability		Cognitive and sensory ability	
	Standardised β	*p*-value	Standardised β	*p*-value	Standardised β	*p*-value
Satisfaction with social network	−0.01	0.851	0.02	0.314	0.03	0.165
Number of family members in social network.	0.09	< 0.001	−0.01	0.917	0.10	0.001
Number of friends in social network.	0.02	0.344	0.02	0.135	−0.01	0.548
Revised UCLA Loneliness Scale.	−0.26	< 0.001	−0.15	< 0.001	− 0.14	< 0.001
*Constant*	(102.24)	*< 0.001*	(120.77)	*< 0.001*	(80.36)	*< 0.001*
R^2^	0.33		0.14		0.19	

All models have been controlled for wealth and socio-demographic variables (age, sex, marital status, educational level and employment status)

For the functional ability component (Table 3), the short R-UCLA loneliness scale (β = − 0.15*, p* < 0.001) was the main SR variable associated with this component. The remaining SR variables were not significant. This model explained 14% of the variance.

For the cognitive and sensory ability component (Table 3) the main SR variable associated was the short R-UCLA loneliness scale (standardised beta β = − 0.14, *p* < 0.001) and having more family members present in their social networks *(*β = 0.10 *p* = 0.001). This model explained 19% of the variance.

## Discussion

The purpose of the study was to describe the SR of a sample of over-50s in Spain and to analyse their association with physical and emotional, functional and cognitive and sensory health variables, which in turn are important elements of healthy ageing. Our results indicate that 67% of the respondents perceived that they did not feel loneliness, which is an important determinant of physical and mental health, as mentioned in several studies [6, 16, 41, 42]. These results are in line with other studies, such as Nyqvist et al., using data from the European Social Survey, where 70% of Spanish participants reported absence of loneliness [43]. Our study uses data from SHARE wave 6, where 95.3% reported having a member of their family present. One explanation to the percentage of respondents that do not feel lonely could be the predominant role of the family in Mediterranean countries and how this cultural context would influence older adults. However, the number of single-person households is progressively increasing and thus, we foresee that loneliness and social isolation in Spanish older people will rise in the future [44].

Among all the social variables, feeling lonely was the variable most closely related to the physical and emotional, functional and cognitive and sensory health components. This is in line with the literature to date, and demonstrates the association of loneliness with important health and well-being outcomes such as functional ability [12, 45], cognitive impairment [46, 47], cardiovascular disease [25, 48], self-perceived health [49] and mortality [13, 42]. These studies help to highlight the importance of feelings of loneliness as we age and the need for good coping skills to fully enjoy these years.

Our study indicates that people who feel more lonely are those with poorer physical and emotional health, results that are in line with those published by other authors [14, 16]. Lower feelings of loneliness were also significantly associated with higher functional ability. This finding is consistent with the work of Lou et al. [45], in which functional ability limitations were associated with loneliness in all age groups. However, Victor and Yang [50] only found this association with young adults. One explanation for this could be that older people might interpret health limitations as normal for their age and therefore accept them better than young adults, for whom poor physical health is unusual. However, the opposite is also possible: people with more functional limitations are impaired from keeping in close contact with friends and family and this in turn would condition them to be less physically active and more disabled.

Similarly, several studies in older adults show that lower feelings of loneliness have been associated with greater cognitive and sensory ability [9, 46, 47], although another study has not identified any significant relationship between these factors [51]. Further studies are needed to determine the extent to which loneliness is associated with cognitive impairment, which is vital for developing effective intervention programmes and policies.

Another SR variable associated with the physical and emotional health component was the number of family members in the network. In this domain, our results suggest that a higher number of family members is associated with better physical and emotional health, whose presence improves health perception, loneliness [16] and lower cognitive impairment [24]. Several studies in southern European countries, including Spain, indicate that the network most frequently found in older adults was the family, rather than other types of relationship (friends, acquaintances, etc.) [52–54]. Accordingly, our results show that 95% of respondents reported having 1–5 family members present in their social network, as opposed to 22% who reported having 1–7 friends. This is evidence of the predominant role of the family in Mediterranean countries and how this cultural context would influence the SR model of older Spaniards. This availability of strong family ties and traditions of family support could be taken into account as a basis for designing future ageing policies, especially in devising courses of action that not only strengthen family ties, but also facilitate support services or flexible working arrangements for those families who want their elders to keep on living with them.

A study in three Mediterranean countries found that a higher level of education is associated with a better perception of health among Spaniards aged 50 and over [55]. Our data show a significant association of the wealth variable with the physical and emotional health components, something that has been supported in the literature for decades [56, 57]. Regarding the cognitive ability component, these approaches should also be revisited in subsequent studies since most of the studies found focus their work on socioeconomic status at birth as significantly influencing cognitive functioning at the end of life [58, 59]. It is well known that the level of education influences cognitive functioning during ageing, such that a higher educational level is a protective factor for cognitive decline, so it is probably a mediating variable in the relationship between the wealth variable and cognitive functioning.

Finally, our results are not only in line with research carried out in the last decade [27, 60] that links the effect of SR with health, but also aims to entwine this view with the effect that SR have on healthy ageing. This dynamic process entails older adults reciprocally benefiting from their SR network and in which they can achieve the desired states of health, less disability or absence of feelings of loneliness. Therefore healthy ageing, health and SR form a complex and indivisible feedback loop.

One of the main strengths of our study is its use of data from a multidisciplinary survey data that features health, psychosocial and economic variables from across Europe. The data are also representative of the Spanish population, so the results can be applied generally to everyone over 50 years old in Spain. In addition, a validated scale has been used to measure one of the main outcome variables, loneliness.

One of the main strengths of our study is the use of data from a survey of a multidisciplinary nature, which incorporates health, psychosocial and economic variables, as well as biological and behaviour variables from across Europe. The data are also representative of the Spanish population, so the results can be generalized to all people over 50 years of age in Spain. In addition, a validated scale has been used to measure one of the main outcome variables, loneliness.

The main limitation is that this study has used cross-sectional data, so no causality conclusions can be drawn. Furthermore, the variance explained by the regression models is not very high, ranging from 14 to 33%. This implies that there must be other explanatory variables not included in the models analysed, which should be considered in future pathways analysis studies. Other methodological limitations were the failure to include other variables such as contacting friends or relatives by digital or remote means, which represent a perspective that has seldom been studied to date. Future research should test possible links between maintaining RS via digital or remote means and health with respect to healthy ageing.

## Conclusions

In conclusion, according to the results of this study, the main determinant of health status, in its physical/emotional, functional and cognitive/sensory aspects, is the feeling of loneliness. Another SR indicator, such as the size of the social network of relatives, has also been related to the same components, except for functional ability.

This study suggests that the SR characteristics (size, satisfaction and intensity measured as the perception of loneliness) that promote older people’s health should be acted upon by encouraging multidisciplinary involvement. Measures could include designing Primary Care schemes for the early detection of groups at risk of loneliness. Facilitating social support work for the loneliest older adults who receive no help from their social networks, and encouraging healthy activities that enable them to strike up and strengthen friendships and other at in each stage of old age, are possible solutions to the problem of loneliness in older adults. Action should also be taken to reinforce both home care strategies and the role of the liaison nurses, not only in managing available resources but also in encouraging families’ involvement and communication to obtain cost-effective and quality results.

Another necessary course of action would be to promote family support programmes (respite care programmes and training for carers) to learn how to care for and look after themselves better. This would not only lead to a good state of health but maintain a healthy and productive relationship between all those involved. In short, it is a question of encouraging social spaces that foster what has always been a hallmark of Spain, namely its extensive social network and its close bonds, mainly family ties.

## Supplementary Information

**Additional file 1: Supplementary Table 1.** Descriptive statistics of the sample variables.

**Additional file 2: Appendix 1.** Description of the Variables.

## Data Availability

Data supporting our findings is available to the scientific community free of charge at www.share-project.org.
