# Supplementary material for: Social relations and health in older people in Spain using SHARE survey data
Source: BMC Geriatr. 2022 Apr 4;22:276. doi: 10.1186/s12877-022-02975-y (PMC8978370; doi:10.1186/s12877-022-02975-y)
Supplement: Supplementary file 1 — Additional file 1: Supplementary Table 1. Descriptive statistics of the sample variables. [file 12877_2022_2975_MOESM1_ESM.docx]

Supplementary Table 1: Descriptive statistics of the sample variables

|  | | Frequency | | Percentage |
| --- | --- | --- | --- | --- |
| Gender | |  | |  |
| Women | | 3076 | | 53.83 |
| Men | | 2507 | | 46.17 |
| Age | |  | |  |
| 50 to 64 years old | | 1946 | | 47.41 |
| 65 to 79 years old | | 2350 | | 34.72 |
| 80 years or more | | 1287 | | 17.87 |
| Marital Status | |  | |  |
| Without a partner | | 1378 | | 24.68 |
| With a partner | | 4205 | | 75.32 |
| Job situation | |  | |  |
| Pensioner | | 2714 | | 49.33 |
| Employed | | 1005 | | 18.27 |
| Housewife | | 1159 | | 21.07 |
| Others | | 624 | | 11.34 |
| Self-rated health | |  | |  |
| Excellent | | 228 | | 4.08 |
| Very well | | 883 | | 15.82 |
| Well | | 2125 | | 38.06 |
| Reasonable | | 1528 | | 27.37 |
| Poor | | 819 | | 14.16 |
| Number of medicines taken | |  | |  |
| 0 to 1 medicines/day | | 2503 | | 44.95 |
| 2 or more medicines/day | | 3066 | | 55.05 |
| Limitation of basic activities of daily living | |  | |  |
| Does not have any limitation | | 4861 | | 88.57 |
| Has some type of limitation (1-7 items) | | 722 | | 11.43 |
| Limitation of instrumental activities of daily living | |  | |  |
| Does not have any limitation | | 4408 | | 82.41 |
| Has some type of limitation (1-9 items) | | 1175 | | 17.60 |
| Degree of satisfaction with social network | |  | |  |
| Low or moderate degree of satisfaction (0 – 8 items) | | 1714 | | 31.41 |
| High degree of satisfaction (9 - 10 items) | | 3372 | | 68.59 |
| Revised UCLA Loneliness Scale | |  | |  |
| Does not feel loneliness | | 3466 | | 67.26 |
| Feels some degree of loneliness (4 – 9 items) | | 1549 | | 32.74 |
| Number of friends in social network | |  | |  |
| No friends | | 4045 | | 77.92 |
| 1 to 7 friends | | 941 | | 22.08 |
| Number of family members in social network | |  | |  |
| No family members | | 281 | | 4.97 |
| 1 to 5 or more | | 4705 | | 95.03 |
|  | Mean | | SD | |
| Age (years) | 70.28 | | 10.55 | |
| Years of education | 8.67 | | 5.11 | |
| Number of chronic pathologies | 1.95 | | 1.62 | |
| Number of medicines | 2.14 | | 1.85 | |
| EURO depression scale (0-12 items) | 2.47 | | 2.62 | |
| Memory problems | 3.30 | | 0.91 | |
| Sensory problems (hearing) | 2.93 | | 0.95 | |
| Sensory problems (sight) | 2.94 | | 0.90 | |
| Physical and emotional health component | 69.08 | | 16.93 | |
| Functional ability component | 90.79 | | 19.25 | |
| Cognitive and sensory ability component | 49.33 | | 16.38 | |
| Level of wealth (€000's) | 220.91 | | 262.60 | |
| Satisfaction with social network (1-10 items) | 8.86 | | 1.28 | |
| Number of family members in social network | 2.14 | | 1.44 | |
| Number of friends in social network | 0.27 | | 0.65 | |
| Revised UCLA Loneliness Scale (3 – 9 items) | 3.72 | | 1.33 | |

Weighted values
