# Supplementary material for: Social relations and health in older people in Spain using SHARE survey data
Source: BMC Geriatr. 2022 Apr 4;22:276. doi: 10.1186/s12877-022-02975-y (PMC8978370; doi:10.1186/s12877-022-02975-y)
Supplement: Supplementary file 2 — Additional file 2: Appendix 1. Description of the Variables. [file 12877_2022_2975_MOESM2_ESM.docx]

**Appendix 1: Description of the Variables**

**1.1 Sociodemographic and socioeconomic variables**

• **Gender**

• **Age** in years

• **Marital status:** What is your marital status? Answer options: married, living with your partner, registered domestic partner, married and living apart from your spouse, single, divorced and widowed. For the analysis, the first two options were grouped in the category “with a partner”, and the rest, as “without a partner”.

• **Years of study: “**How many years have you been studying full time?” The answer option is a scale ranging from 0 to 25 years maximum.

• **Employment situation:** “In general, which of the following options best describes your current employment situation?” Response options are the following: retiree, employee, housewife or others that includes the unemployed, people with disability benefits and in another situation.

• **Socioeconomic status:** It was calculated by adding the value variables of the main residence; value of any other real state; value of bank accounts; bonds, stocks and mutual funds; value of sharing part of a business and value of automobiles; and subtracting the mortgage on the principal residence and financial obligations.

**1.2 Health Components**

**1.2.1 Physical and emotional health**

**• Number of medications:** The respondent is provided with a list of 15 medications, consumed at least once a week, related to various pathologies (for example, medications for cholesterol, hypertension or diabetes, etc.) and the option to complete additional ones if it does not match those mentioned.

• **Number of chronic diseases**: Calculated from a list of 30 common chronic diseases such as heart attacks, diabetes, stroke, cancer, cataracts, among others.

**• EURO-D depression scale:** Consisting of 12 items (fatigue, loss of appetite, insomnia, loss of concentration, loss of interest, irritability, dysphoric mood, crying, anhedonia, pessimism, guilt, and ideas of death ). The type of answer is dichotomous (yes / no). The total score is made by adding the positive scores. The maximum score that can be obtained is 12, indicative of severe depression. It is considered as a case of depression if a score greater than or equal to 4 is obtained.

**• Self-perceived health:** The US version of the scale is used for this variable. In answering the question: “Would you say your health is ... ?” Respondents rate their current overall health on a five-point Likert scale (excellent, very good, good, fair, poor). The difference between the European version and the US version is the range of response options. The response categories in the European version range from "very good" to "very poor" and those in the US version, which are based on the SF-36 questionnaire range from "excellent" to "poor".

**1.2.2 Functional capacity**

**Basic Activities of Daily Living (ABVD):** “Please tell me if you have any difficulties with the following due to a physical, mental, emotional or memory problem? Exclude any difficulties that you expect to last less than three months”. The answer options are: get dressed, even put on shoes and socks; walk through a room; take a bath or take a shower; eating, like cutting food; get in or out of bed; use the bathroom, including getting up or downstairs; none of these". SHARE uses the modified version that includes 6 activities. The type of response is dichotomous (yes / no) for each of the functions. The total score is made by adding the affirmative scores. The score ranges from 0 to 6. The higher the score, the more difficulties the person has to carry out these activities and the lower the mobility.

**• Instrumental activities of daily living (AIVD):** “Please tell me if you have any difficulty with the following due to a physical, mental, emotional or memory problem? Exclude any difficulties that you expect to last less than three months.” The answer options are: use a map to find out how to get around in a strange place; prepare a hot meal; buy groceries, make phone calls, take medicine, work around the house or garden; manage money, such as paying bills and tracking expenses; leave the house independently and access transportation services, wash personal clothes; none of those. SHARE uses the modified version that includes 7 activities. The type of answer is dichotomous (yes / no) for each of the functions. The total score is made by adding the affirmative scores. The score ranges from 0 to 7. The higher the index, the more difficulties the person has to carry out these activities and the lower the mobility.

**1.2.3 Cognitive and sensory capacity**

**• Sensory hearing problems:** This variable is evaluated with two questions: “how is your hearing?”, rated on a five-point Likert scale (excellent, very good, good, fair, poor); and “do you usually wear a hearing aid?” with a yes / no answer.

**• Sensory vision problems:** This variable is evaluated with two questions: “how good is your vision to see things from a distance, such as recognizing a friend across the street?”; and “How good is your vision to see things up close, such as reading printed newspapers?” Both are rated on a five-point Likert scale (excellent, very good, good, fair, poor).

**• Memory problems:** “How would you rate your memory at this time?” Respondents rate it on a five-point Likert scale (excellent, very good, good, fair, bad).

**1.3 Variables of social relations**

**1.3.1 Number of family and friends on social networks**

The information for these two variables is extracted from the question: “In the last 12 months, who are the people with whom you talked most frequently about important things, or are important to you for any reason?” The respondent can provide a list of up to seven people, which are called “contacts in the respondent's social network” and their response is taken as the basis for the following question regarding the degree of satisfaction with the people mentioned.

**1.3.2 Degree of satisfaction with social networks**

The question that this variable represents refers to the degree of interaction that the respondent has with the people in his social network, talking about matters that are important to him and with some confidence. Overall satisfaction with the aforementioned people who make up your network is measured on a single global scale that ranges from 0 (totally dissatisfied) to 10 (completely satisfied). This variable, along with the number of family members and friends, are aggregate variables, which are based on the combination of information from all members of the social network mentioned by the respondent, so they are configured as lost if any of the members of the red has missing information about this variable and they are coded separately.
